# The role of *Bacillus acidophilus* in osteoporosis and its roles in proliferation and differentiation

**DOI:** 10.1002/jcla.23471

**Published:** 2020-08-11

**Authors:** Chen Chen, Baokang Dong, Yuming Wang, Qiang Zhang, Bangmao Wang, Shuzhi Feng, Yu Zhu

**Affiliations:** ^1^ Department of Geriatric Tianjin Medical University General Hospital Tianjin China; ^2^ Department of Orthopedics Tianjin First Center Hospital Tianjin China; ^3^ Department of Gastroenterology Tianjin Medical University General Hospital Tianjin China; ^4^ Department of Clinical Laboratory Tianjin Haihe Hospital Tianjin China

**Keywords:** gut microbiota, osteoblasts, osteoclasts, osteoporosis

## Abstract

**Background:**

Osteoporosis is one of the most closely related diseases associated with the elderly. In recent years, the studies found that gut microbiota can cause osteoporosis. We evaluated the role of Bacillus acidophilus in osteoporosis and its roles in proliferation and differentiation.

**Methods:**

We selected 5 healthy people and 10 osteoporosis patients and analyzed their level of 25‐hydroxyvitamin D and procollagen type I N‐terminal peptide (PINP), the characteristic of gut microbiota. The effect of *lactobacillus acidophilus and Lactobacillus rhamnosus* supernatant and butanoic acids on proliferation, differentiation, and maturity of osteoblasts MC3T3‐E1 and osteoclasts RAW 264.7 cells and the activity of alkaline phosphatase, concentration of osteocalcin, and the expression of RUNX2, RANK, NFATc1, cathepsin K, DC‐STAMP, OSCAR, WNT2, and CTNNB1 were measured in the above cell lines.

**Results:**

The diversity of gut microbiota in osteoporosis patients is decreased and imbalanced with lower abundance of lactobacillus and butyric acid bacteria; meanwhile, 25‐hydroxyvitamin D and PINP of osteoporosis patient were significantly lower than the normal group. The proliferation, differentiation, and maturity of MC3T3‐E1 cells were stimulated; the activity of alkaline phosphatase, concentration of osteocalcin, and the expression of RUNX2, NFATc1, cathepsin K, DC‐STAMP, OSCAR, WNT2, and CTNNB1 were improved by supernatant of *lactobacillus acidophilus*, *Lactobacillus rhamnosus* and butanoic acids; however, the proliferation, differentiation, maturity, and the expression of RANK, NFATc1, cathepsin K, DC‐STAMP, OSCAR, WNT2, and CTNNB1 in RAW 264.7 cells were suppressed.

**Conclusions:**

The *lactobacillus acidophilus* and *Lactobacillus rhamnosus* supernatant could stimulate the proliferation, differentiation, and maturation of osteoblasts; the production of butyric acid may be the potential mechanism.

## Graphical abstract

The diversity of gut microbiota is decreased and imbalance with lower abundance of lactobacillus and butyric acid bacteria in osteoporosis patients. The gut microbiota regulate the proliferation, differentiation, and maturation of osteoblasts, and the production of butyric acid may be the potential mechanism.

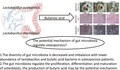

## 1 INTRODUCTION

As a mammal, humans have an incredibly large number of microbes living on and inside our bodies and a complex community of symbiotic bacteria.^1^ These microorganisms play an important role in the physiological activities of the human body. The largest microecosystem in the human body is the intestinal tract.^2^

In recent years, increasing evidence has accumulated, suggesting that the human gut microbiota can be thought of as a very important organ acquired after birth.^3^ New attention has been directed at the relationship between the gut microbiota and human health. The interaction between the gut microbiota and the host is mutualistic, as the host can provide a suitable environment and nutrients to support bacterial growth while the gut microbiota plays important roles in food digestion, the production of vitamins and other nutrients, and increasing resistance against invasion by foreign pathogens.^4^, ^5^ In addition to these important roles, the microbiota can also affect many physiological functions of the human body. Under normal circumstances, the intestinal microecosystem remains relatively stable, and this balance is beneficial to the human body. However, a variety of factors (such as drugs, alcoholism, mental factors, gastrointestinal surgery, radiation therapy, and aging) can alter this balance, resulting in the development of a variety of diseases, such as multiple enteritis, diabetes, asthma, obesity, osteoporosis, and metabolic syndrome. ^6^, ^7^, ^8^, ^9^, ^10^

Osteoporosis is a disease intimately linked to aging.^11^ It is a common bone disease characterized by decreased bone mass and bone degeneration. Recent studies have shown that the gut microbiota is associated with decreased bone mass and the pathogenesis of osteoporosis.^12^ These microorganisms may alter the relative activities of osteoclasts and osteoblasts through different pathways, such as metabolite production and altering of the host metabolism and immune system, both of which can affect bone metabolism.

Therefore, the goals of this study were to analyze the changes in the gut microbiota in osteoporosis patients and to study the effects of the metabolites of two probiotics, *Lactobacillus acidophilus* and *Lactobacillus rhamnosus* (LGG), on an osteoblast precursor cell line (MC3T3‐E1) and on an osteoclast precursor cell line (RAW 264.7). By studying the relationship between the gut microbiota and osteoporosis and investigating the mechanism by which the gut microbiota affects bone metabolism from the perspective of osteoblast‐mediated bone formation and osteoclast‐mediated bone resorption, this study could lay a theoretical foundation for further investigation into these topics and provide data to support the use of probiotics as a clinical intervention for osteoporosis.

## 2 MATERIALS AND METHODS

### 2.1 Research subjects

#### 2.1.1 Selection of research subjects

Ten patients with osteoporosis and osteoporotic fractures who were hospitalized in the Department of Geriatrics of General Hospital of Tianjin Medical University from January 2017 to December 2017 were randomly recruited into the osteoporosis group. Five healthy people without osteoporosis were randomly recruited for the control group. The general data of normal and osteoporosis group are shown in Table 1. All participants in the experiments were matched for age and gender. This study was approved by the Medical Ethics Committee of Tianjin Medical University General Hospital on 2015 according to the Declaration of Helsinki, and patients provided informed consent before the experiments.

**Table 1:** General data of normal and osteoporosis group

	Age (years)	Systolic pressure (mm Hg)	Diastolic pressure(mm Hg)	BMI (kg/m^2^)
Control	90.14 ± 6.07	128.86 ± 16.87	61.86 ± 8.51	22.47 ± 2.40
Osteoporosis	88.50 ± 5.40	129.25 ± 22.05	60.13 ± 9.37	23.79 ± 2.19
*P* value	All > .05			

The inclusion criteria for the osteoporosis group included (a) patients who were diagnosed with osteoporosis by dual‐energy X‐ray absorptiometry or with osteoporotic fractures by imaging and (b) nonviolent fractures.

The exclusion criteria were as follows: (a) patients with fractures caused by violence or trauma and (b) patients with other bone diseases, such as osteomalacia, renal osteodystrophy, or other metabolic bone diseases or bone tumors.

#### 2.1.2 Analysis of the demographics data of the selected subjects

The sex, age, height, weight, and blood pressure of each selected patient were collected to calculate the mean age, mean body mass index (BMI), mean systolic blood pressure (SBP), and mean diastolic blood pressure (DBP). Fasting blood samples were obtained to test the serum levels of total protein (TP), albumin (ALB), alanine aminotransferase (ALT), aspartate aminotransferase (AST), alkaline phosphatase (ALP), serum creatinine (CREA), blood urea (UREA), blood uric acid (UA), fasting blood glucose (FPG), blood calcium (Ca), blood phosphorus (P), blood potassium (K), blood sodium (Na), triglycerides (TG), total cholesterol (TC), low‐density lipoprotein cholesterol (LDL), 25‐hydroxyvitamin D, osteocalcin (OC), parathyroid hormone (PTH), procollagen type I N‐terminal peptide (PINP), and C‐terminal telopeptide of type I collagen (CTX).

#### 2.1.3 Measurement of bone density and diagnosis of osteoporosis

The medical staff of the Bone Densitometry Section of the Department of Geriatrics at the General Hospital of Tianjin Medical University used dual‐energy X‐ray absorptiometry (Lunar DPX Prodigy, GE Healthcare, USA) to measure the bone density of the 2nd to the 5th lumbar vertebrae (L2‐L4) and the hip (left femoral neck, trochanter, and Ward's triangle) of the patients in each group. The total bone density (g/cm^2^) was calculated to obtain T‐scores. The diagnostic criteria for osteoporosis were based on T‐scores ≤−2.5 in any of the following sites: lumbar vertebrae, femoral neck, or total hip.

### 2.2 Fecal DNA extraction and testing

Stool specimens were collected from the patients in the osteoporosis and healthy control groups using a sterile specimen container. Approximately 1 gram of feces was placed in the sterile stool specimen container, and the container was immediately sealed and stored in liquid nitrogen. The samples were sent to the laboratory within 1 hour for storage in a −80°C freezer. A cador Pathogen 96 QIAcube HT Kit (Qiagen, USA) was used to extract bacterial genomic DNA from the stool samples. The concentrations of the genomic DNA samples (in 2 µL) were measured with an ultraviolet‐visible spectrophotometer. Pure DNA should exhibit an obvious absorption peak at an optical density of 260 nm (OD_260_) with an OD_260_/OD_280_ ratio of approximately 1.8.

Construction of a high‐throughput sequencing library and Illumina‐based sequencing using a MiSeq instrument was performed by GENEWIZ (Suzhou, China). The sequencing library was constructed using the MetaVx^TM^ library construction kit (GENEWIZ, Inc, South Plainfield, NJ, USA). Custom polymerase chain reaction (PCR) primers were used to amplify two highly variable regions of the bacterial 16S rDNA gene (the V3 and V4 regions) using 30‐50 ng of DNA as the template. The V3 and V4 regions were amplified using a forward primer with the sequence “CCTACGGRRBGCASCAGKVRVGAAT” and a reverse primer with the sequence “GGACTACNVGGGTWTCTAATCC.” Library quality was evaluated using Agilent 2100 Bioanalyzer (Agilent Technologies), and the library concentrations were determined using a Qubit 2.0 Fluorometer (Invitrogen, Carlsbad, CA). After the DNA library was mixed, double‐end sequencing (PE, 2 × 300 bp) was performed using an Illumina MiSeq (Illumina) instrument according to manufacturer's instructions, and the sequence data were analyzed with MiSeq Control Software (MCS) provided by MiSeq.

### 2.3 Cell culture

Mice osteoblasts MC3T3‐E1 were purchased from the Tianjin Institute of Orthopaedics and Traumatology and cultured in α‐MEM with 10% fetal bovine serum (Gibco; Thermo Fisher Scientific, Inc) at 37˚C and 5% CO_2_. Mice osteoclasts RAW 264.7 were obtained from the Tianjin Medical University and cultured in DMEM (High Glucose) with 10% fetal bovine serum (Gibco; Thermo Fisher Scientific, Inc, Waltham, MA, USA), penicillin (100 U/ml), and streptomycin (100 mg/mL) at 37°C and 5% CO_2_.

### 2.4 Bacterial culture and supernatant extraction

Cryogenic vials containing the *lactobacillus acidophilus* (LABS) and *Lactobacillus rhamnosus* (LGG) solution stored in a liquid nitrogen tank were removed and completely thawed at room temperature. One milliliter of the bacterial solution was pipetted into 100 mL of the prepared De Man, Rogosa, and Sharpe (MRS) medium in a sterile laminar flow workbench, and the inoculated culture dishes were incubated at 37°C. After 72 hours, the bacterial growth was stable and the subsequent experiments were carried out. Culture medium containing LABS and LGG was transferred to a centrifuge tube and centrifuged in a high‐speed refrigerated centrifuge at 14 000 *g* for 10 minutes at 4°C. After centrifugation, the supernatant was filtered through a membrane (0.22 μm) 3 times to obtain bacterial supernatant of LABS or LGG.

### 2.5 Osteoblast and osteoclast proliferation

MC3T3‐E1 and RAW 264.7 cells (2.5 × 10^3^ per well) treated with bacterial culture supernatant (LABS or LGG, mixed at ratios of 1:20, 1:50, and 1:100) or sodium butyrate (0.5 mM) were inoculated in 96‐well plates and incubated for 48 hours. Then, 20 μL of tetrazolium dye (MTT) solution (5 mg/mL) was added to each well, and the plates were incubated at 37°C in 5% CO_2_ for 4 hours. The medium was aspirated, and 100 μL of dimethyl sulfoxide (DMSO) was added to the wells. The plates were then stirred for 10 minutes. After the crystals were completely dissolved, the OD values were measured at 490 nm using a microplate reader. Phosphate‐buffered saline (PBS) was as control and added into 96‐well plates.

### 2.6 Determination of ALP activity and OCN

MC3T3‐E1 cells were inducted by induction medium which contained vitamin C (50 mg/L) and β‐sodium glycerophosphate (10 mM) or uninduction medium which did not contain vitamin C and β‐sodium glycerophosphate, and LABS bacterial culture supernatant mixed at a ratio of 1:20 and 0.5 mM sodium butyrate medium were collected after induction for 7 days, respectively. PBS was as control and added into plates. The media were centrifuged for 10 minutes at 1500 r/min, and the supernatants were collected. The alkaline phosphatase (ALP) activity and osteocalcin (OCN) concentration were measured by ELISA according to the manufacturer's instructions.

### 2.7 Real‐time PCR assay

MC3T3‐E1 and RAW 264.7 cells are treated in induction medium. MC3T3‐E1 cells are inducted by vitamin C (50 mg/L) and β‐sodium glycerophosphate (10 mM), RAW 264.7 cells are inducted by 50 μg/L RANKL, and LABS bacterial culture supernatant is mixed at a ratio of 1:20 or 0.5 mM sodium butyrate medium. PBS was as control and added into plates. The total RNA of MC3T3‐E1 and RAW 264.7 cells was extracted using the TRIzol reagent (Invitrogen) according to the manual's instructions. Then cDNA was obtained by oligo‐dT primers or stem‐loop reverse transcriptase (RT) primers by RT‐PCR Kit (Takara Bio Inc), and mRNA expression of the target genes was detected using real‐time PCR assay (Applied Biosystems 7500 Real‐Time PCR). The RNA was used as a template for complementary DNA (cDNA) synthesis with the following conditions: denaturation at 95℃ for 10 min, followed by 40 cycles at 95℃ for 15s, 60℃ for 60s, 60℃ for 15s, and a final elongation at 95°C for 15 seconds. RT‐qPCR was performed to detect nuclear factor of activated T‐cell cytoplasmic 1 (NFATC1), cathepsin K, dendritic cell–specific transmembrane protein (DC‐STAMP), osteoclast‐associated receptor (OSCAR), wingless‐type MMTV integration site family member 2 (WNT2), β‐catenin gene (CTNNB1), and receptor activator for nuclear factor‐κ B (RANK), and GAPDH was used as the internal reference gene. The quantified results were calculated using the 2^−ΔΔCq^ method.^13^ The full details of the primers used in these experiments are shown in Table 2

**Table 2:** Quantitative real‐time polymerase chain reaction primers in the experiments

Gene	Primers
*NFATc1*	Forward: 5′‐TGGGAGATGGAAGCAAAGAC‐3′
	Reverse: 5′‐ATAGAAACTGACTTGGACGGG‐3′
*cathepsin K*	Forward: 5′‐ATGTGGGTGTTCAAGTTTC‐3′
	Reverse: 5′‐TCAATGCCTCCGTTCT‐3′
*DC‐STAMP*	Forward: 5′‐AAAACCCTTGGGCTGTTCTT‐3′
	Reverse: 5′‐AATCATGGACGACTCCTTGG‐3′
*OSCAR*	Forward: 5′‐CCTAGCCTCATACCCCCAG‐3′
	Reverse: 5′‐CGTTGATCCCAGGAGTCACAA‐3′
*WNT2*	Forward: 5′‐ CTCGGTGGAATCTGGCTCTG‐3′
	Reverse: 5′‐CACATTGTCACACATCACCCT‐3′
*RANK*	Forward: 5′‐CTCTATGCCCGTGTCCCCTGAAAA‐3′
	Reverse: 5′‐GGCCGCGATGTCCCGTCCTT‐3′
*CTNNB1*	Forward: 5′‐ATGGAGCCGGACAGAAAAGC‐3′
	Reverse: 5′‐CTTGCCACTCAGGGAAGGA‐3′
*GAPDH*	Forward: 5′‐AGGTCGGTGTGAACGGATTTG‐3′
	Reverse: 5′‐TGTAGACCATGTAGTTGAGGTCA‐3′

### 2.8 Effects of bacterial supernatant on osteoblast and osteoclast culturing and induction

MC3T3‐E1 cells are inducted by vitamin C (50 mg/L) and β‐sodium glycerophosphate (10 mM), and RAW 264.7 cells are inducted by 50 μg/L RANKL. The extracted LABS and LGG bacterial supernatant was mixed at a ratio of 1:20 to generate bacterial supernatant culture medium and 0.5 mM sodium butyrate (represent butanoic acids) in which MC3T3‐E1 and RAW 264.7 cells were incubated at 37°C in 5% CO_2_. The bacterial supernatant culture medium and sodium butyrate were replaced every 48 hours. PBS was as control and added into plates. Above induction medium is refreshed every 3 days.

ALP/Alizarin Red staining was performed on the MC3T3‐E1 cells after 7 days of incubation in induction medium. Two milliliters of citric acid concentrate was added to 100 mL of deionized water and then mixed with acetone (2:3 ratio) to produce a fixing solution. The prepared fixing solution was added to the culture plates to fix the osteoblasts for 3 minutes. Substrate was applied dropwise to completely cover the fixed osteoblasts, and the plates were incubated at 37°C for 15 minutes in the dark before being washed with distilled water for 5 minutes. The cells were stained with Alizarin Red solution for 7 minutes and then washed with distilled water for 5 minutes. The slides were dried at room temperature and sealed. The cells were observed and photographed under a microscope.

Tartrate‐resistant acid phosphatase (TRAP) staining of RAW 264.7 osteoclasts was performed after treating them with the same method for 5 days.

### 2.9 Luciferase reporter assay

MC3T3‐E1 and RAW 264.7 cells are treated in induction medium. MC3T3‐E1 cells are inducted by vitamin C (50 mg/L) and β‐sodium glycerophosphate (10 mM), RAW 264.7 cells are inducted by 50 μg/L RANKL, and LABS bacterial culture supernatant is mixed at a ratio of 1:20 or 0.5 mM sodium butyrate medium. PBS was as control and added into plates.

Then cells were transfected using the Transfection Reagent (Biomiga) following the manufacturer's instructions. Cells were co‐transfected with AP‐1 promoter‐luciferase plasmids and pRL‐SV40 (Promega) as an internal control for 6 h. The luciferase reporter assay was performed using the Dual‐Luciferase Reporter Assay System (Promega) on Berthold TriStar LB 941 (Berthold Technologies).

### 2.10 Statistical analysis

Data were represented by mean ± SD and analyzed by SPSS 11.0 software. The one‐way ANOVA and Tukey's post hoc analysis were used for general measurement data. R software (version, 2.15.3) was used for nonmetric multidimensional scaling (NMDS) analysis. *P* < .05 was defined as a significant difference.

## 3 RESULTS

### 3.1 Comparison of the general data between the groups

No statistically significant differences were observed between the two groups upon comparison of the biochemical parameters (ALB, ALT, AST, ALP, CREA, UREA, UA, FPG, Ca, P, K, Na, TG, TC, LDL, and PTH) in Table 3, indicating that secondary osteoporosis caused by common reasons, such as endocrine diseases and nutritional factors, could be excluded. Moreover, the patients had no history of treatment with glucocorticoids, corticosteroids, antiepileptic drugs, antineoplastic drugs (eg, methotrexate), or heparin. The levels of two bone turnover markers, 25‐hydroxyvitamin D and PINP, in the osteoporosis group were significantly lower than those in the control group; however, there were no obvious abnormalities in the CTX and OCN between the two groups (Table 4). These features indicate a lack of active bone resorption in the osteoporotic patients with high bone turnover activity, thus demonstrating that the patients enrolled in the groups suffered from senile osteoporosis with low bone turnover activity. Therefore, these patients could be diagnosed with primary osteoporosis and met the criteria to be included in the subsequent experiments.

**Table 3:** Clinic biochemistry data of normal and osteoporosis group

	Control	Osteoporosis	*P* value
TP (g/L)	67.43 ± 6.13	65.75 ± 3.85	>.05
ALB (g/L)	34.57 ± 2.94	34.25 ± 2.66	
ALT (U/L)	11.57 ± 3.46	15.63 ± 8.91	
AST (U/L)	17.57 ± 4.79	18.88 ± 8.08	
ALP (U/L)	74.14 ± 7.65	77.13 ± 44.08	
TBIL (μM)	8.57 ± 3.22	8.05 ± 2.35	
UREA (mM)	5.43 ± 2.74	7.49 ± 6.01	
CREA (μM)	70.43 ± 15.60	69.13 ± 36.37	
URIC (μM)	281.71 ± 75.60	271.86 ± 123.11	
GLU (mM)	4.97 ± 0.53	5.03 ± 0.58	
Ca (mM)	2.26 ± 0.96	2.30 ± 0.15	
PHOS (mM)	0.98 ± 0.15	1.00 ± 0.11	
K (mM)	4.31 ± 0.56	4.13 ± 0.39	
Na (mM)	140.86 ± 1.35	139.63 ± 2.77	
TC (mM)	4.32 ± 0.77	4.66 ± 1.09	
TG (mM)	1.32 ± 0.33	1.65 ± 0.70	
LDL (mM)	2.86 ± 0.65	2.94 ± 0.77	
PTH (pM)	7.20 ± 3.19	5.24 ± 2.70	

**Table 4:** Vitamin D and bone turnover marker of normal and osteoporosis group

	25‐OH Vitamin D (nM)	OC (ng/mL)	CTX (ng/mL)	PINP (ng/mL)
Control	50.17 ± 13.59	21.36 ± 7.13	0.47 ± 0.21	78.78 ± 34.22
Osteoporosis	26.51 ± 11.39	19.6 ± 4.59	0.68 ± 0.27	41.83 ± 13.16
*P* value	<.05	>.05	>.05	<.05

### 3.2 Changes in the gut microbiota of osteoporosis patients

**Figure 1:**
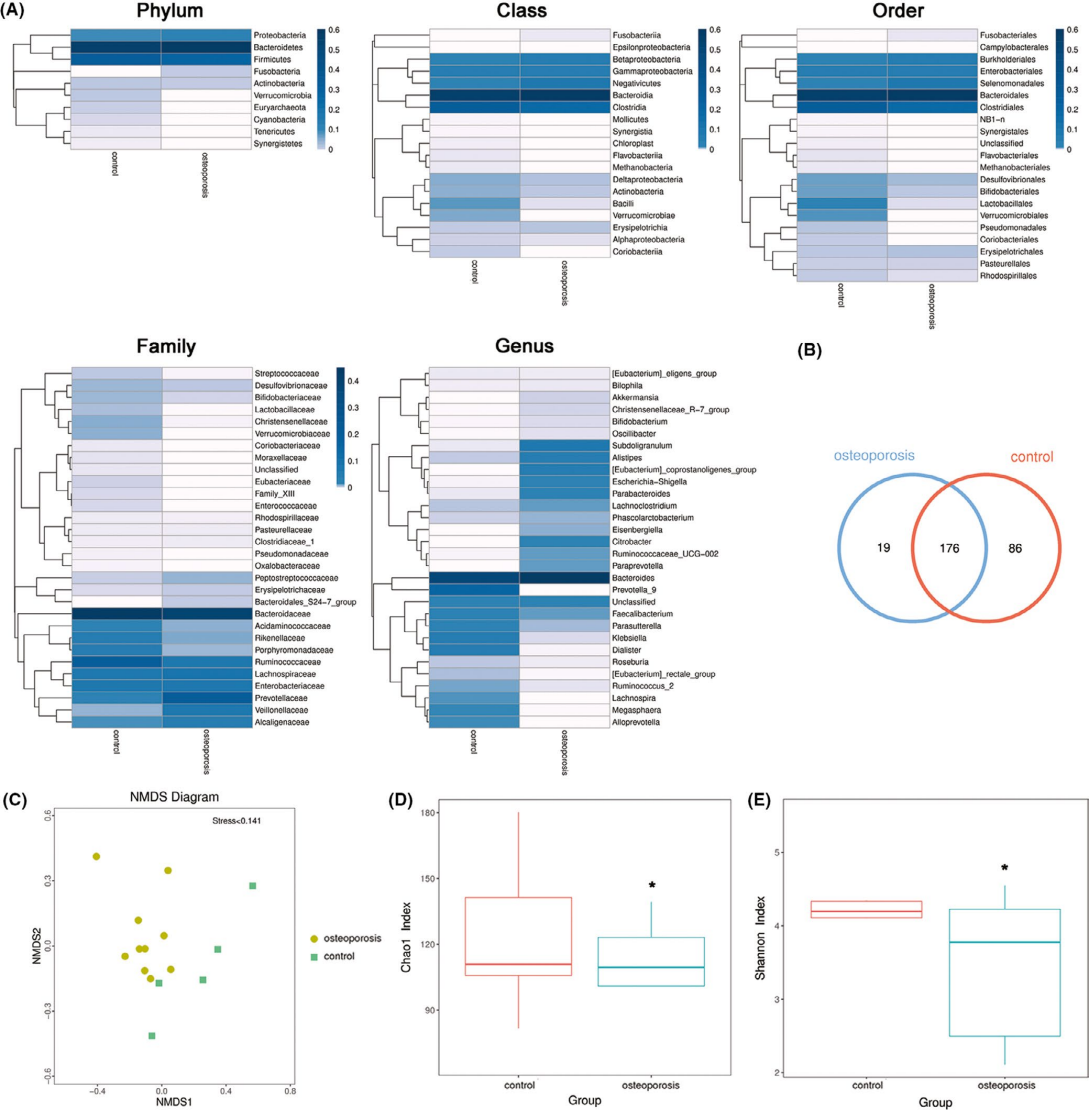
Characteristics of gut microbiota of healthy controls and osteoporosis patients. A, The heatmap of gut microbiota of healthy controls and osteoporosis patients in different classification. B, Venn diagram shows the changed gut microbiota OTU in healthy controls and osteoporosis patients. C, The NMDS analysis shows the heterogeneity of intestinal microbiota in the healthy controls and osteoporosis patients. D, The Chao1 index and E, Shannon index show the diversity in intestinal microbiota of osteoporosis patients compared with healthy control. ^*^, compared with control group, *P* < .05

High‐throughput sequencing was used to analyze and compare the 16S rRNA sequences present in the gut microbiota of the osteoporosis patients and healthy controls. The structure and characteristic of the gut microbiota in the osteoporosis patients were altered (Figure 1). Especially, the levels of *Lactobacillus* and butyric acid‐producing bacteria were decreased, and abundance of pathogenic bacteria, such as *Clostridium*, was increased.

### 3.3 Effects of LABS, LGG, and sodium butyrate on the proliferation of osteoblasts and osteoclasts

**Figure 2:**
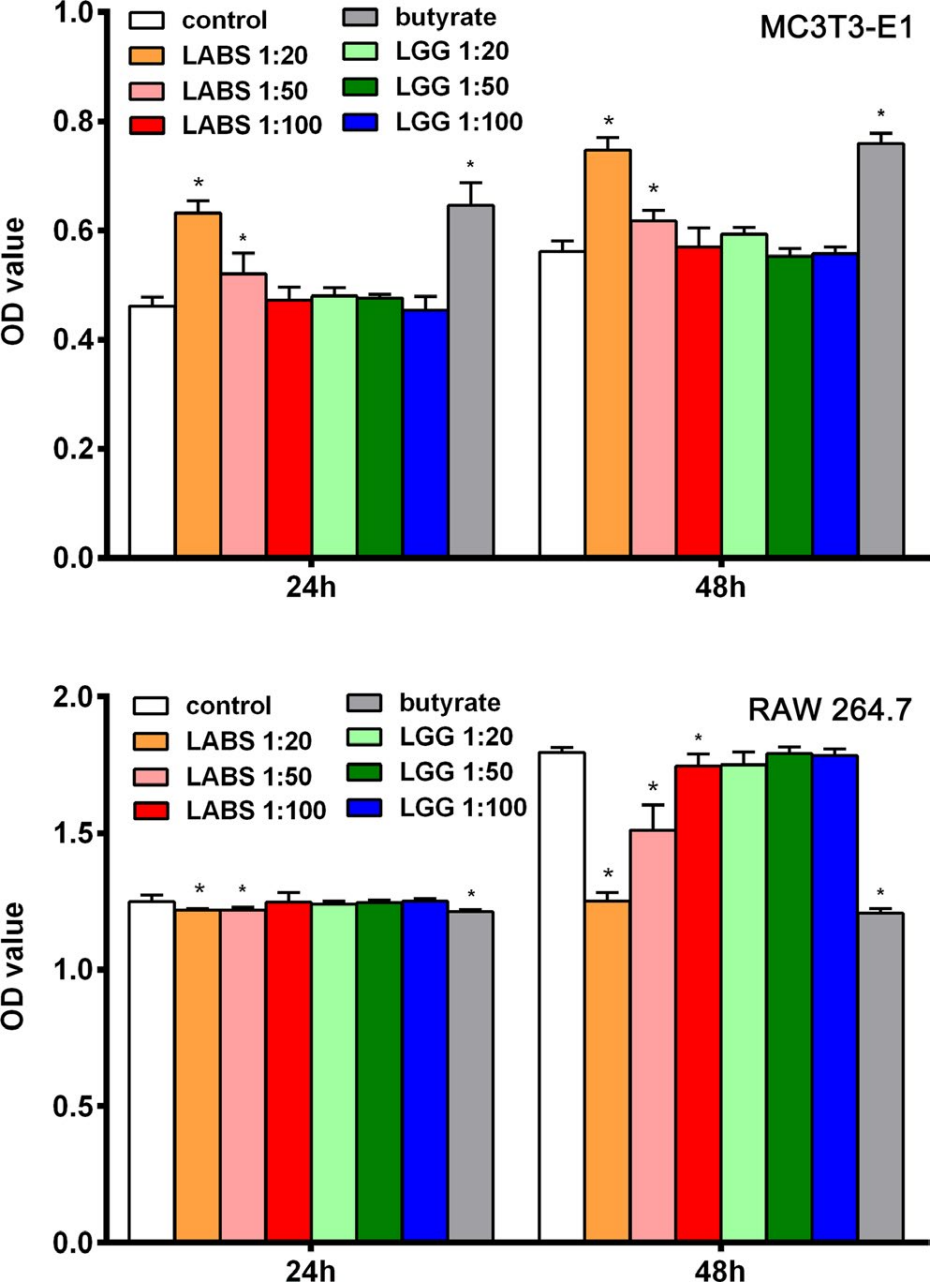
Effects of gut microbiota on proliferation in osteoblasts and osteoclasts. MTT assay results showed the proliferation of osteoblast MC3T3‐E1 and osteoblast RAW 264.7 co‐cultured with supernatant of LABS and LGG, and sodium butyrate for 24 and 48 h. ^*^, *P* < .05 compared with control group

There is a significantly enhanced proliferation of osteoblast MC3T3‐E1 after incubation in LABS supernatant (1:20 and 1:50) for both 24 and 48 h; meanwhile, LGG supernatant did not enhance the proliferation on any ratio for 24 and 48 hours. There is a significantly reduced proliferation of osteoclast RAW 264.7 after incubation in LABS supernatant (1:20 and 1:50) for 24 and the ratio of 1:20, 1:50, and 1:100 for 48 hours; however, LGG supernatant could not reduce the proliferation by any ratio for both 24 and 48 hours. Sodium butyrate (0.5 mM) could stimulate the proliferation of osteoblast MC3T3‐E1 and significantly inhibit the proliferation of osteoclast RAW 264.7 for 24 and 48 hours (Figure 2).

### 3.4 Effects of LABS, LGG, and sodium butyrate on differentiation‐related factors in osteoblasts and osteoclasts

**Figure 3:**
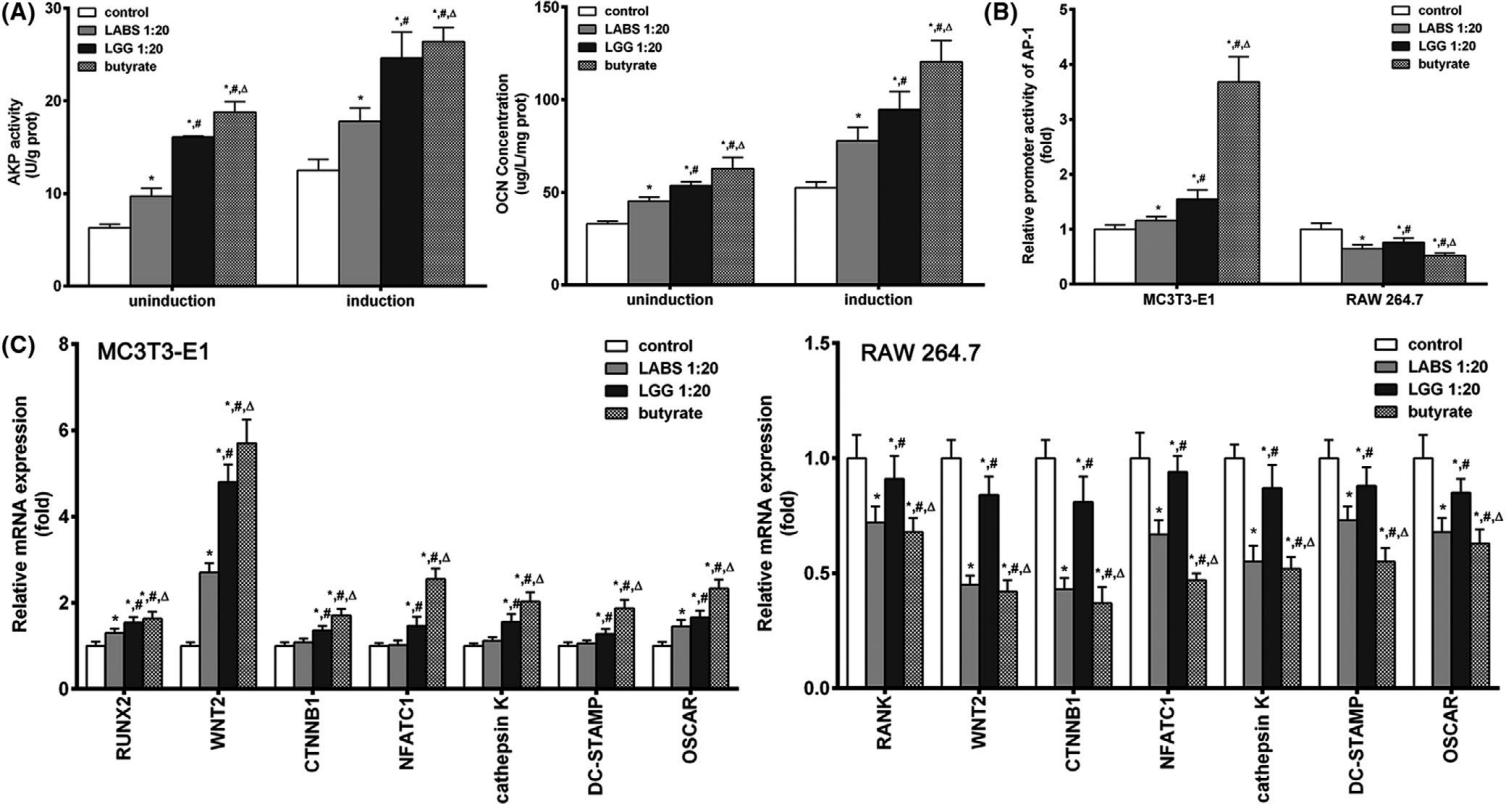
Effects of gut microbiota on differentiation‐related factors in osteoblasts and osteoclasts. A, The effect of supernatant of LABS and LGG, and sodium butyrate on ALP activity and OCN concentration in osteoblasts MC3T3‐E1. B, The effect of supernatant of LABS and LGG, and sodium butyrate on the relative promoter activity of AP‐1 in osteoblasts MC3T3‐E1 and osteoclasts RAW 264.7. C, The effect of supernatant of LABS and LGG, and sodium butyrate on the expression of osteoblast and osteoclast‐related gene in osteoblasts MC3T3‐E1 and osteoclasts RAW 264.7. ^*^, *P* < .05 compared with control group; ^#^, *P* < .05 compared with LABS group; ^△^, *P* < .05 compared with LGG group

ALP and OC are markers of osteoblast differentiation and play important roles in bone formation. The higher the ALP activity and OCN expression, the higher the osteogenic activity of the osteoblasts.^14^ After 7 days, there were significantly higher ALP activity and OCN concentration by LABS supernatant (1:20), LGG supernatant (1:20), or 0.5 mM sodium butyrate treatment of osteoblasts MC3T3‐E1 in induction and uninduction medium; meanwhile, the ALP activity and OCN concentration were higher in induction compared to uninduction medium (Figure 3A).

AP‐1 was the promoter of protein expression of c‐fos and considered to play important roles in stimulating osteoblasts and inhibiting osteoclast. In this study, we found that the relative activity of AP‐1 was significantly increased in osteoblasts MC3T3‐E1 incubated in media containing LABS supernatant (1:20) or 0.5 mM sodium butyrate and significantly decreased in osteoclasts RAW 264.7 by luciferase reporter assay (Figure 3B).

Runt‐related transcription factor 2 (RUNX2) is a specific osteoblast factor and is closely linked to osteoblast function. RANK plays an important role in bone resorption and is highly expressed on the surface of osteoclasts. It participates in osteoclast differentiation, thus leading to bone loss. NFATc1, cathepsin K, DC‐STAMP, OSCAR, WNT2, and CTNNB1 stimulate osteoblasts and inhibit osteoclast. The mRNA expression of NFATc1, cathepsin K, DC‐STAMP, OSCAR, RUNX2, WNT2, and CTNNB1 was significantly increased in osteoblasts MC3T3‐E1 incubated in media containing LABS supernatant (1:20) or 0.5 mM sodium butyrate. The expression levels of the mRNAs of RANK, NFATc1, cathepsin K, DC‐STAMP, OSCAR, WNT2, and CTNNB1 significantly decreased in osteoclasts RAW 264.7 (Figure 3C).

### 3.5 Effects of LABS, LGG, and sodium butyrate on the maturity of osteoblasts and osteoclasts

**Figure 4:**
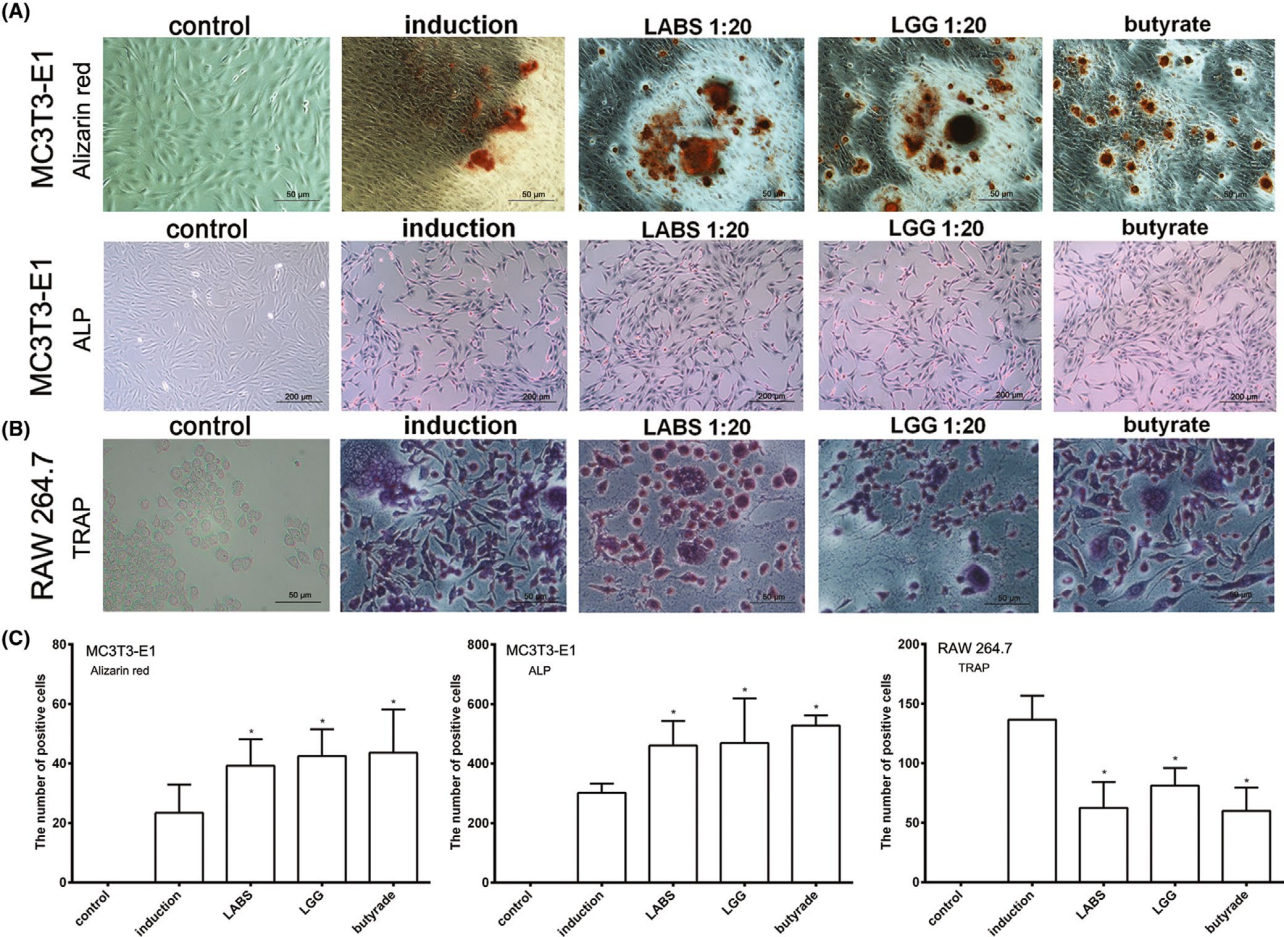
Effects of gut microbiota on the maturity of osteoblasts and osteoclasts. A, The effect of supernatant of LABS and LGG, and sodium butyrate on dark staining of the osteoblasts MC3T3‐E1 cell membrane and cytoplasm after ALP/Alizarin Red staining. B, The effect of supernatant of LABS and LGG, and sodium butyrate on the cytoplasmic volume and additional nuclei in osteoclasts RAW 264.7 after TRAP staining. C, The graph of gut microbiota on the ALP, Alizarin Red, and TRAP staining in osteoblasts MC3T3‐E1 and osteoclasts RAW 264.7 cell. ^*^, *P* < .05 compared with induction group

After ALP/Alizarin Red staining, dark staining of the cell membrane and cytoplasm was observed. The results showed that the number of darkly stained cells (maturity osteoblasts) increased in the osteoblasts MC3T3‐E1 cultured in media containing LABS supernatant (1:20), LGG supernatant (1:20) and sodium butyrate (0.5 mM). The MC3T3‐E1 was cultured in induction medium as positive control (Figure 4A).

Following culturing with LABS supernatant (1:20), LGG supernatant (1:20), and sodium butyrate (0.5 mM), the RAW 264.7 cells gradually differentiated into osteoclasts, that is, osteoclasts gradually formed after 5 days of induction, as indicated by increased cytoplasmic volume and additional nuclei. The results showed that the number of darkly stained cells increased in osteoblasts cultured and induced in media containing LABS supernatant (1:20) or butyric acid (Figure 4B).

## 4 DISCUSSION

The aging population and age‐related bone damage have developed into major public health problems in China.^15^ Osteoporosis patients are not only increasing in number but also have increased risk of suffering from additional senile diseases.^16^ Osteoporotic fractures in these patients result in difficulties in treatment and care and can also cause many economic and social problems.^17^ Therefore, research on novel effective treatment methods and the mechanisms underlying senile osteoporosis is important, as it has practical and important implications.

Recent studies have found that the gut microbiota is associated with decreased bone mass and the pathogenesis of osteoporosis. These microorganisms may affect bone metabolism by altering the relative activities of osteoclasts and osteoblasts via different pathways, such as those involving bacterial metabolites and others affecting host metabolism and immunity.^18^, ^19^, ^20^ Although the gut microbiota varies widely, the composition of the gut microbiota in older people significantly shifts from obligate anaerobic bacteria to facultative anaerobic bacteria. In particular, the abundance of pathogenic proteobacteria increases, while that of anti‐inflammatory lactobacilli decreases. These changes lead to increased risk of inflammation and, in turn, mediate the differentiation and maturation of osteoclasts. This process indicates that the characteristic changes in the gut microbiota of older people may increase their risk of osteoporosis. In this study, analyses of the fecal microflora from osteoporosis patients and healthy control subjects revealed dysbacteriosis, decreased levels of lactobacilli and some butyric acid‐producing bacteria, and increased levels of pathogenic clostridium in the osteoporosis patients.

Lactobacillus is one of the most widely studied probiotics, and the term “lactobacillus” is actually a generic name for any bacteria that can produce large amounts of lactic acid from fermentable carbohydrates.^21^ In this study, LABS and LGG, which belong to the genus *Lactobacillus*, were selected for study. The well‐accepted roles of these two bacterial species include maintaining the balance of the gut microbiota, promoting defecation, reducing the occurrence of constipation, improving immunity, lowering blood lipid levels, promoting nutrient absorption, improving lactose intolerance, and imparting anticancer effects. An increased proportion of probiotics, such as lactobacilli, can increase the levels of short‐chain fatty acids, resulting in a positive effect on bone metabolism. Butyric acid, a short‐chain fatty acid, can directly affect bone cells and inhibit the growth of osteoclasts in vitro and can stimulate the proliferation and differentiation of osteoblasts.

The results of our experiments showed that the number of darkly stained cells increased in osteoblasts cultured and induced in media with LABS supernatant (1:20) or butyric acid. ALP/Alizarin Red staining can make cell membrane and cytoplasm appear darkly stained. Therefore, this finding may indicate that the most mature and abundant osteoblasts were induced and differentiated in the medium with LABS supernatant (1:20). This observation suggests that LABS metabolites may contain short‐chain fatty acid that can directly stimulate osteoblasts. Furthermore, the levels of osteoblast stimulation were positively related to the metabolite concentration and the duration of incubation. In contrast, no significant increase in osteoblast activities was observed in the medium containing LGG supernatant, indicating that there were no short‐chain fatty acids among its metabolites that could directly affect osteoblasts. It is known that LABS can produce butyric acid, but it is not clear whether LGG shares this trait. In light of the observation of the increased number of darkly stained osteoblasts after culturing and induction in medium containing 0.5 mM sodium butyrate, we conclude that LABS stimulates osteoblast proliferation, differentiation, and maturation of via butyric acid in its metabolites, thereby improving bone quality.

Butyric acid, a short‐chain fatty acid, induces cell morphology changes and cell differentiation in different cell types.^22^ However, the direct effects of this short‐chain fatty acid on bone cells have not been well studied. In recent years, studies have shown that ALP and OCN are expressed throughout the processes of proliferation and differentiation in MC3T3‐E1 cells. ALP and OCN are markers of osteoblast differentiation and play important roles in bone formation. It has been reported that butyric acid can increase ALP activity and OCN expression. This observation indicates that butyric acid can stimulate the activity of MC3T3‐E1 cells to promote osteoblast proliferation and differentiation. This finding has been confirmed in this study. ALP activity and OCN expression were significantly increased after incubation with LABS supernatant (1:20) or 0.5 mM sodium butyrate medium for 7 days, indicating that LABS promotes osteoblast differentiation and maturation via butyric acid production.

RUNX2 is associated with osteoblast differentiation. Its main function is to positively regulate osteoblast function and to stimulate ALP to promote bone maturation.^23^ RANK binds to the receptor activator for nuclear factor‐κB ligand (RANKL) released by osteoblasts. During bone resorption, osteoclast precursor cells constantly differentiate into osteoclasts, and RANK is highly expressed on the surface of the osteoclast precursor cells. RANK binds to the RANKL released by osteoblasts to produce downstream signals that increase the expression levels of many transcription factors in osteoclast precursor cells to activate the Wnt/β‐catenin pathway.^24^ The Wnt/β‐catenin and AP‐1/NFATc1 pathway are important signaling cascade that promotes osteoblast formation and inhibits osteoclast formation. Wnt protein and β‐catenin are encoded by the WNT2 and CTNNB1 genes, respectively.^25^ In this study, treatment with LABS supernatant or butyric acid upregulated the mRNA expression levels of NFATc1, cathepsin K, DC‐STAMP, OSCAR, RUNX2, WNT2, CTNNB1, and RANK in MC3T3‐E1 osteoblasts and downregulated the expression levels of the above genes in RAW 264.7 osteoclasts.

c‐fos, which plays an important role in osteoblast and inhibits osteoclast formation, is a part of AP‐1. We found that the relative promoter activity of AP‐1 was changed with the same tendency. These results confirm that LABS supernatant and butyric acid can promote the differentiation and maturation of osteoblasts and inhibit the proliferation and differentiation of osteoclasts by regulating the expression levels of the above genes.

The experimental results showed that a high concentration of LABS supernatant could stimulate the proliferation, differentiation, and maturation of osteoblasts. The aforementioned effects were not present in cells exposed to LGG supernatant. LABS can produce butyric acid, which can stimulate osteoblast proliferation and differentiation, while LGG does not produce butyric acid. Based on these observations, we speculated that LABS may stimulate osteoblast proliferation, differentiation, and maturation via butyric acid production.

## AUTHOR CONTRIBUTIONS

CC and BD contributed equally to this work. YZ and BW conceived and designed the study. YW, QZ, and SF performed the experiments and analyzed the data. YZ wrote the manuscript. BW reviewed and modified the manuscript.

## ETHICAL APPROVAL

This study was approved by the Ethics Committee of Tianjin Medical University General Hospital. Guarantor: Yuming Wang.
